# A198 BIO-EXPERIENCED ULCERATIVE COLITIS PATIENTS REQUIRING ADMISSION HAVE A TWO-FOLD RISK OF COLECTOMY AS COMPARED TO BIO-NAÏVE PATIENTS

**DOI:** 10.1093/jcag/gwae059.198

**Published:** 2025-02-10

**Authors:** A Panteluk, K Chappell, M Gozdzik, K Kroeker

**Affiliations:** University of Alberta Faculty of Medicine & Dentistry, Edmonton, AB, Canada; University of Alberta Faculty of Medicine & Dentistry, Edmonton, AB, Canada; University of Alberta Faculty of Medicine & Dentistry, Edmonton, AB, Canada; University of Alberta Faculty of Medicine & Dentistry, Edmonton, AB, Canada

## Abstract

**Background:**

Admission rates for patients with ulcerative colitis (UC) are decreasing, however patients admitted with a UC flare are at increased risk of colectomy. In recent years, numerous advanced therapies have emerged to treat flares and maintain remission. Many patients are now “bio-experienced”, defined as prior/current exposure to at least one advanced therapy. In flare management, colectomy is reserved for patients with severe or refractory disease, or to manage complications such as toxic megacolon or perforation. It is not known if a patient’s bio-exposure status (i.e. bio-experienced or bio-naive) affects their risk of requiring a colectomy when they are hospitalized for a UC flare. Such information is important to clinicians when choosing to initiate therapy, determining the timing of therapy escalation and allowing patients to understand their risk to make informed treatment decisions.

**Aims:**

To assess the relative risk of 90-day colectomy in bio-experienced versus bio-naïve patients admitted to hospital with UC flare as well as secondary outcomes such as time to first advanced therapy in hospital and length of stay (LOS).

**Methods:**

This is a single centre retrospective cohort study that included patients admitted with a diagnosis of UC flare to a major tertiary hospital in Edmonton, AB from Nov 2019 to Apr 2024. The cohort was designated as bio-naïve, having never received advanced therapies or bio-experienced, having received at least one advanced therapy prior to admission. Administrative data extraction and chart review was used to obtain the primary and secondary outcomes. Relative risk of colectomy was calculated between groups and a 95% confidence interval generated for this. The secondary outcomes were analyzed using an unpaired *t*-test with the significance level of *p<0.05*. For LOS with colectomy, given significant variance, median and IQR were reported instead.

**Results:**

In total, there were 216 admissions; 87 patients were bio-experienced and 129 were bio-naïve at the time of admission. Twenty-six patients underwent colectomy within 90 days. Of the patients who were bio-experienced, 18.39% underwent colectomy compared to 7.75% in the bio-naïve group. The relative risk of 90-day colectomy in the bio-experienced compared to bio-naïve patients was 2.37 (1.13-4.98 95% CI).

Secondary outcomes showed similar average length of stay, however bio-experienced patients were placed on advanced therapy sooner and those requiring colectomy had shortened LOS, compared to bio-naïve patients (Figure 1). Chart review for other secondary outcomes (eg. prior therapies, disease duration) is underway.

**Conclusions:**

In this retrospective cohort study, we demonstrated that the relative risk of colectomy for UC patients admitted with flare is 2.3 fold higher in bio-experienced compared to bio-naïve patients.

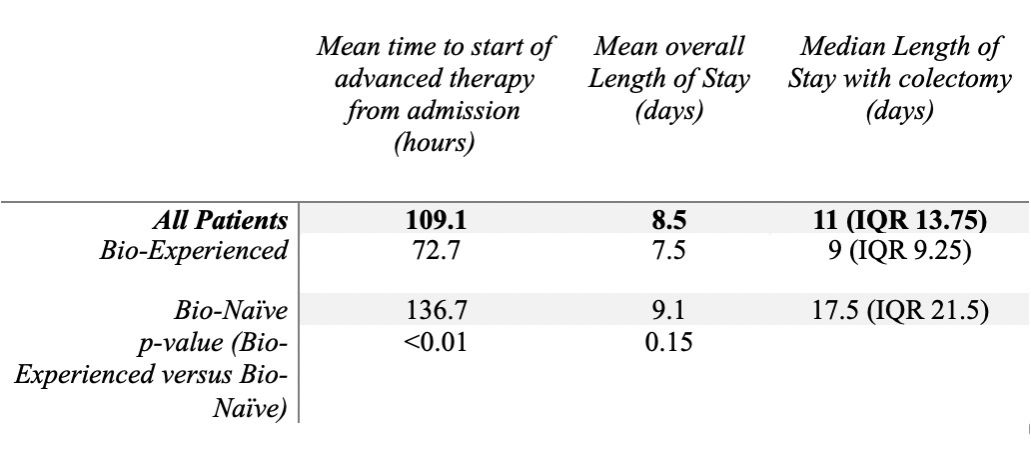

Figure 1

**Funding Agencies:**

None

